# Contributions of EEG-fMRI to Assessing the Epileptogenicity of Focal Cortical Dysplasia

**DOI:** 10.3389/fncom.2017.00008

**Published:** 2017-02-20

**Authors:** Francesca Pittau, Lorenzo Ferri, Firas Fahoum, François Dubeau, Jean Gotman

**Affiliations:** ^1^Department of Neurology and Neurosurgery, Montreal Neurological Institute and Hospital, McGill UniversityQuébec, QC, Canada; ^2^Neurology Department, Geneva University HospitalsGeneva, Switzerland

**Keywords:** EEG-fMRI, FCD, neuronal migration disorder, epileptic network, localization

## Abstract

**Purpose:** To examine the ability of the BOLD response to EEG spikes to assess the epileptogenicity of the lesion in patients with focal cortical dysplasia (FCD).

**Method:** Patients with focal epilepsy and FCD who underwent 3T EEG-fMRI from 2006 to 2010 were included. Diagnosis of FCD was based on neuroradiology (MRI+), or histopathology in MRI-negative cases (MRI−). Patients underwent 120 min EEG-fMRI recording session. Spikes similar to those recorded outside the scanner were marked in the filtered EEG. The lesion (in MRI+) or the removed cortex (in MRI−) was marked on the anatomical T1 sequence, blindly to the BOLD response, after reviewing the FLAIR images. For each BOLD response we assessed the concordance with the spike field and with the lesion in MRI+ or the removed cortex in MRI−. BOLD responses were considered “concordant” if the maximal *t*-value was inside the marking. Follow-up after resection was used as gold-standard.

**Results:** Twenty patients were included (13 MRI+, 7 MRI−), but in seven the EEG was not active or there were artifacts during acquisition. In all 13 studied patients, at least one BOLD response was concordant with the spike field; in 9/13 (69%) at least one BOLD response was concordant with the lesion: in 6/7 (86%) MRI+ and in 3/6 (50%) MRI− patients.

**Conclusions:** Our study shows a high level of concordance between FCD and BOLD response. This data could provide useful information especially for MRI negative patients. Moreover, it shows in almost all FCD patients, a metabolic involvement of remote cortical or subcortical structures, corroborating the concept of epileptic network.

## Introduction

Focal cortical dysplasia (FCD), a frequent cause of drug resistant focal epilepsy, is a neuronal migration disorder characterized by abnormalities of the laminar structure of the cortex, variably associated with cytopathological features including giant (or cytomegalic) neurons, dysmorphic neurons, and balloon cells (Palmini et al., 2004). The classification of FCD was reviewed by an ILAE task force (Blumcke et al., 2011), which proposes three categories: FCD type I characterized by radial (FCD type Ia) or tangential (FCD type Ib) dyslamination of the neocortex; FCD type II refers to a cortical dyslamination and dysmorphic neurons without (type IIa) or with balloon cells (type IIb); and FCD type III occurs in combination with other types of lesion (hippocampal sclerosis in type IIIa, tumors in type IIIb, vascular malformations in type IIIc, and epileptogenic lesions acquired in early life in type IIId). Structural MRI can detect dysplastic changes in a variable percentage of cases and more easily in patients exhibiting balloon cells (type IIb) (Tassi et al., 2012); 3T MRI fails to localize the dysplastic lesion in up to 87% of patients with FCD type I and 33% of those with FCD type II (Bernasconi et al., 2011). Surgical results are variable (Palmini et al., 1991; Kloss et al., 2002; Tassi et al., 2002; Chassoux et al., 2012; Guerrini et al., 2015) and depend on the ability to delineate and fully excise the entire region of dysplastic cortex (Paolicchi et al., 2000; Wagner et al., 2011). The lesion may be small and difficult to detect on routine MRI (De Ciantis et al., 2016), parts of the dysplastic tissue may be undetectable (Bernasconi et al., 2011), or additional dysplastic cortical areas remote from the lesion exist (Fauser et al., 2009), all reasons that explain the variability of the surgical outcomes (Fauser et al., 2015).

Recording simultaneously functional MRI (fMRI) and EEG is a noninvasive method detecting cerebral hemodynamic changes related to interictal epileptic discharges (IEDs) on scalp EEG. Several studies demonstrated the ability of EEG-fMRI to characterize various forms of focal and generalized epilepsy (Gotman et al., 2005; Laufs and Duncan, 2007; Gotman, 2008) and the clinical utility of this approach (Zijlmans et al., 2007; Moeller et al., 2009; Pittau et al., 2012; An et al., 2013). Previous small series of FCD patients studied with EEG-fMRI (Federico et al., 2005; Tyvaert et al., 2008) showed that focal IEDs are associated with metabolic changes (IEDs-related BOLD activations or deactivations) in the lesion itself and the overlying cortex but also in areas and neural networks extending beyond the FCD. A larger study of patients with type II FCD (Thornton et al., 2011) comparing EEG-fMRI with intracranial EEG findings, confirmed that EEG-fMRI provides useful information on the extension and epileptogenicity of FCDs and helps to identify patients with good surgical outcome (those with discreet and solitary lesion and well localized epileptogenic area) from patients who would be less likely to benefit from resective surgery (those with remote epileptogenic areas).

The aim of our study is to further characterize EEG-fMRI changes in a population of patients with focal cortical dysplasia demonstrated by MRI or histopathology. More specifically, we wish to assess if EEG-fMRI can define the localization and extension of the FCD, and if it can contribute to determine the localization and extension of the epileptogenic area(s) explained by the primary dysplastic lesion.

## Methods

### Subjects

We included consecutive patients with a diagnosis of focal epilepsy and FCD who underwent EEG-fMRI from October 2006 to July 2010. The diagnosis of FCD was based on the neuroradiological findings (MRI+) and when applicable confirmed by histopathology or, in case of negative MRI (MRI−), on histopathology reports only. Some patients had surgery and resections were guided by the integration of clinical and laboratory findings excluding the EEG-fMRI results. The study was approved by the institutional research ethics board. Each subject gave written informed consent in accordance with the Research Ethics Committee of the Montreal Neurological Institute and Hospital.

### EEG-fMRI acquisition

EEG was continuously recorded inside a 3T MRI scanner (Siemens, Trio, Germany). No sedation was given. The EEG acquisition was performed with 25 MR compatible electrodes (Ag/AgCl) placed on the scalp using the 10-20 (reference at FCz) and the 10-10 (F9, T9, P9, F10, T10, and P10) placement systems. Two additional electrodes were placed to record the electrocardiogram. The head of the patient was immobilized with a pillow filled with foam microspheres (Siemens, Germany) to minimize movement artifacts and for patient's comfort. Data were transmitted from a Brain Amp amplifier (Brain Products, Munich, Germany, 5 kHz sampling rate) to the EEG monitor located outside the scanner room via an optic fiber cable.

A T1-weighted anatomical acquisition was first done (1 mm slices, 256 × 256 matrix, TE = 7.4 ms, TR = 23 ms, flip angle 30°) and used to superimpose functional images. The functional data were acquired in runs of 6 min each with the patient in the resting state using a T2^*^-weighted EPI sequence (64 × 64 matrix; either 25 slices, 5 × 5 × 5 mm, TE = 30 ms, TR = 1.7 s, or 33 slices, 3.7 × 3.7 × 3.7 mm, TE = 25 ms, TR = 1.9 s; flip angle 90°). The data are available upon request to the authors.

### EEG-fMRI processing

#### EEG

Brain Vision Analyser software (Brain Products, Munich, Germany) was used for off-line correction of the gradient artifact (Allen et al., 2000). A 50-Hz low-pass filter was also applied to remove the remaining artifact. The ballistocardiogram artifact was removed by independent component analysis (Bénar et al., 2003). A neurologist reviewed the EEG recording and marked IEDs, according to those observed during clinical monitoring (outside the scanner).

#### fMRI

The EPI images were motion corrected and smoothed (6 mm full width at half maximum) using the software package from the Brain Imaging Center of the Montreal Neurological Institute (http://www.bic.mni.mcgill.ca/software/). Data were then analyzed as an event-related design using fMRIstat software (Worsley et al., 2002). The EPI frames were realigned using a linear 6-parameter rigid-body transformation (3 translations and 3 rotations) to correct for movement effects. To account for residual movement artifacts, the six parameters used for the realignment were also integrated in the analysis as confound regressors in the general linear model. A regressor for each type of IED was built using the timing and duration of each event and convolved with four hemodynamic response functions (HFRs) with peaks at 3, 5, 7, and 9 s (Bagshaw et al., 2004). All these regressors were included in the same general linear model. A statistic t map was obtained for each regressor using the other regressors as confounds (a study was performed for each type of interictal event) in the fMRI analysis (fMRIstat) (Worsley et al., 2002). At each voxel, the maximum *t*-value was taken from the four individual t maps created with the four HRFs. To be significant, a response needed to have a spatial extent threshold of five contiguous voxels having a *t* > 3.1 corresponding to *p* < 0.05, corrected for multiple comparisons (Family Wise Error rate = FWE) resulting from the number of voxels in the brain and the use of four HRFs. The t map results were represented using red-yellow scale corresponding to positive BOLD changes (activation) and blue-white scale to negative BOLD changes (deactivation). BOLD responses related to each IED type were reviewed by three experts; responses outside the brain parenchyma were ignored.

### EEG-fMRI analysis

For each patient, the analysis proceeded as follows:

#### EEG and BOLD

The spike field (the region thought to generate the spike) was estimated at the sub-lobar level by visual inspection of the scalp EEG. For example, spikes seen at electrodes F7-T3-T5 corresponded to the anterior and mid aspect of left temporal lobe, or spikes generated at Fp1-F3-F7 corresponded to the anterior aspect of left frontal lobe. Each BOLD response (positive and negative) for each type of IED was considered “concordant” if the max *t*-value corresponded to the localization of the spike field determined by EEG. If a patient had both activation and deactivation, we selected the response with the highest *t*-value. We considered “not concordant” a BOLD response with a max *t*-value outside the spike field. We reported also the BOLD responses in subcortical areas and in areas far from the spike field, to allow the description of the correlated epileptic network.

#### BOLD and lesion (MRI positive or resected cortex in MRI negative)

To allow the co-registration between the BOLD response and the lesion or the resected cortex, firstly we had to mark the lesion or the resected cortex in the anatomical T1 sequence acquired during the EEG-fMRI session. These markings were performed using MNI-Display software (http://www.bic.mni.mcgill.ca/software/Display/Display.html); they were made by a trained physician blind to the EEG-fMRI results, after revising the FLAIR images acquired separately from the EEG-fMRI session. The concordance between the lesion and the marked area were judged independently and blindly with respect to the BOLD results by two neurologists. Then, the co-registration between the marking and the EEG-fMRI results was made with Anatomist (http://brainvisa.info/download.html). Each BOLD response (positive and negative) for each type of IED was considered “concordant” if the maximal *t*-value overlapped with the marking. For MRI—we considered concordant also a distance up to 1 cm away from the border of the marking, because of the possible post-surgical brain displacement.

To estimate the extent of the BOLD response compared to the anatomical lesion, we computed, for the MRI positive patients, the volume of the lesion and the volume of the BOLD cluster (with *t* > 3.1) concordant with the lesion.

## Results

### Subjects

Twenty patients (10 males) with FCD and refractory focal epilepsy underwent EEG-fMRI studies. Seven were excluded from the analysis because of the lack of IEDs (*n* = 5) or artifacts (*n* = 2) during scanning. Therefore, we studied 13 subjects, all with definite FCD. Mean age at study was 30 years (range, 11–50). Mean age at seizure onset was 8.2 years (range, 1–17). Clinical details of these patients are described in Table 1.

**Table 1:** **Clinical characteristics of the 13 patients showing IEDs during EEG-fMRI**.

**Patients**	**Age/Sex/A.O**.	**Semiology**	**Interictal EEG**	**Ictal EEG**	**MRI (FCD location)**	Intracranial electrodes^*^	**Resection**	**Histopathology**	**Follow-up Outcome**
1	32/F/4	Fear sensation. R arm and leg stiffness, then GTCS.	C3 spikes	N.A.	Middle and inferior left frontal gyri and premotor cortex	No	None	–	
2	34/M/6	Diffuse stiffness and abrupt fall.	C3-P3-Pz polyspikes	F3C3FzCz rhythmic discharge	L precuneus	No	None	–	
3	26/F/8	Hearing changes, not verbally responsive, oral automatism, sometimes eyes deviation to right. Few GTCS.	Low amplitude spikes max at F3	Rhythmic slow activity max at F3,F7	L middle frontal gyrus, over opercular region	LCA, LCM, LLES, LOF, LH; epileptic generator seen in LLES	Anterior part of L middle frontal gyrus	FCD IIb	12 month Engel 1
4	23/F/12	Cephalic aura, head and eyes deviation to the L, GTCS.	Spike and slow wave equipotential at F8-T4 and F10-T10 with bilateral diffusion	2 Hz bilateral diffuse spike and wave discharge	R orbito-frontal	No	None	–	
5	30/M/ 1	Nocturnal. Panic sensation and hyperkinetic seizure.	Bil fronto-temporal regions bursts of rhythmic delta activity	Fp2-Fp1-F4-F8 sharp waves	R fronto-polar region	No	R fronto-polar region	FCD IIb	12 month Engel 3
6	50/F/3	Epilepsia partialis continua, contractions of the R side of the face. “Absence” episodes.	C3 spikes	Bilateral diffuse discharge	L precentral gyrus	No	None	–	
7	33/M/7	Sensory aura in R arm, then not tonic elevation of arm.	C3-P3-O1-Pz spikes	N.A.	L sup parietal lobule	No	None	–	
8	19/M/16	R arm tickling sensation, R arm jerks, then GTCS.	C3-P3-Cz spikes	attenuation of background activity	Normal	8X8 grid over frontocentral parietal convexity, activity over contacts 22-23 (post central gyrus)	L superior pre-central gyrus	FCD IIb	12 month Engel 4
9	25/F/14	Vocalizations, grimaces, then tonic posturing.	Low voltage Fp2-F8 spikes	F10, T10, F8, T4 rhythmic dischrage	Normal	RA, RH, RPH, RIM, ROF, epileptic generator in lateral ROF	R lateral orbito-frontal	FCDIIa	30 month Engel 1
10	22/M/3	Throat squeezing sensation, diffuse stiffening.	F3 >F4 spikes	Bil F rhythmic spike and waves	Normal	LOF, ROF, LAC, RAC, LFP, RFP, LSC, epileptic generator seen over LSC	L superior F gyrus	FCD IIb	30 month Engel 4
11	11/M/5	Cephalic sensation, head deviation toward the L, some GTCS.	F4 spikes	F4 rhythmic discharge	Normal	No	R mesial second frontal gyrus	FCDIIa	-
12	41/M/6	Staring, L hemibody clonic movements, loss of consciousness and post ictal confusion.	1) T6P10 spikes; 2) low voltage sharp activity over R frontal	R > L frontal low voltage fast activity	Normal	RT, RA, RIP, ROF, RSMA, RAC, RMC, RPC, RH, RPH, epileptic generator seen over RH and RPH	R SMA and mid cingulate cortex	FCD Ia	25 month Engel 4
13	48/F/3	Chilling sensation on the R arm, then R shoulder stiffness and tonic posture (R>L). Rare GTCS.	Widespread discharges over L hem with max at T3 or over L temporal	Low amplitude rhythmic delta in anterior regions	Normal	LSMA, LAC, LSC, LOF; activity seen over LOF	1) remote L SMA resection; 2) extended resection of L pre-motor cortex max at the level of the second frontal gyrus	FCD IIb	12 month Engel 4

* *All intracerebral depth electrodes except for pt. 8. A.O.: age (in years) at onset; FCD, focal cortical dysplasia; GTCS, general tonic-clonic seizure; L, left; LAC, left anterior cingulate; LASMA, left anterior supplementary motor area; LC, left cingulate; L-E, left epidural electrode; LFP, left frontal-polar; LLES, electrode aiming the left lesion; LH, left hippocampus; LMC, left mid cingulate; LOF, left orbito-frontal; LPC, left posterior cingulate; LPSMA, left posterior supplementary motor area; LSC, left superior cingulate; LSMA, left supplementary motor area; R, right; RA, right amygdala; RAC, right anterior cingulate; RASMA, right anterior supplementary motor area; RC, right cingulate; RFP, right fronto-polar; RH, right hippocampus; RIM, right mid insular cortex; RIP, right posterior insular cortex; RLES, right lesional electrode; RMC, right mid cingulate; ROF, right orbito-frontal; RPC, right posterior cingulate; RPH, right posterior hippocampus; RPSMA, right posterior supplementary motor area; RSC, right superior cingulate; RT, right temporal*.

In seven patients FCD was detected by MRI: in six the MRI was characteristic for a transmantle FCD and in one (#4) a thickness and blurring of the cortex was associated with a suspicious sulcus appearance (Bernasconi et al., 2011). Two MRI+ patients were operated and the histopathological analysis showed the presence of FCD type IIb. In six MRI− patients, FCD was detected by histopathological analysis of the surgical sample. This analysis showed the presence of FCD type IIb in 3 cases, IIa in 2 cases, and Ia in 1 case.

Six patients underwent invasive EEG investigations, one MRI+ and five MRI− (Table 1). The dysplastic lesion was frontal or fronto-central in 11 cases and parietal in two.

Eight patients were operated and type 1 was found in one (MRI−), type IIa in two (both MRI −) and type IIb in five (2 MRI+ and 3 MRI−).

Concerning post-surgical follow-up, two patients (1 MRI+ and 1 MRI−) were in Engel 1; 5 were in Engel 3 or 4 (1 MRI+, 4 MRI−). Follow up was not available for patient 11.

### EEG-fMRI data

Of the 13 patients with active EEG during acquisition, 10 patients had a single type of IED and three patients had two types of IED, so 16 analyses were computed. In 14 analyses IEDs were unilateral, in one they were bilateral and in one they were bilateral and diffuse. The number of events recorded during the fMRI session ranged from 4 to 459 (average 90, median 29).

**Figure 1:**
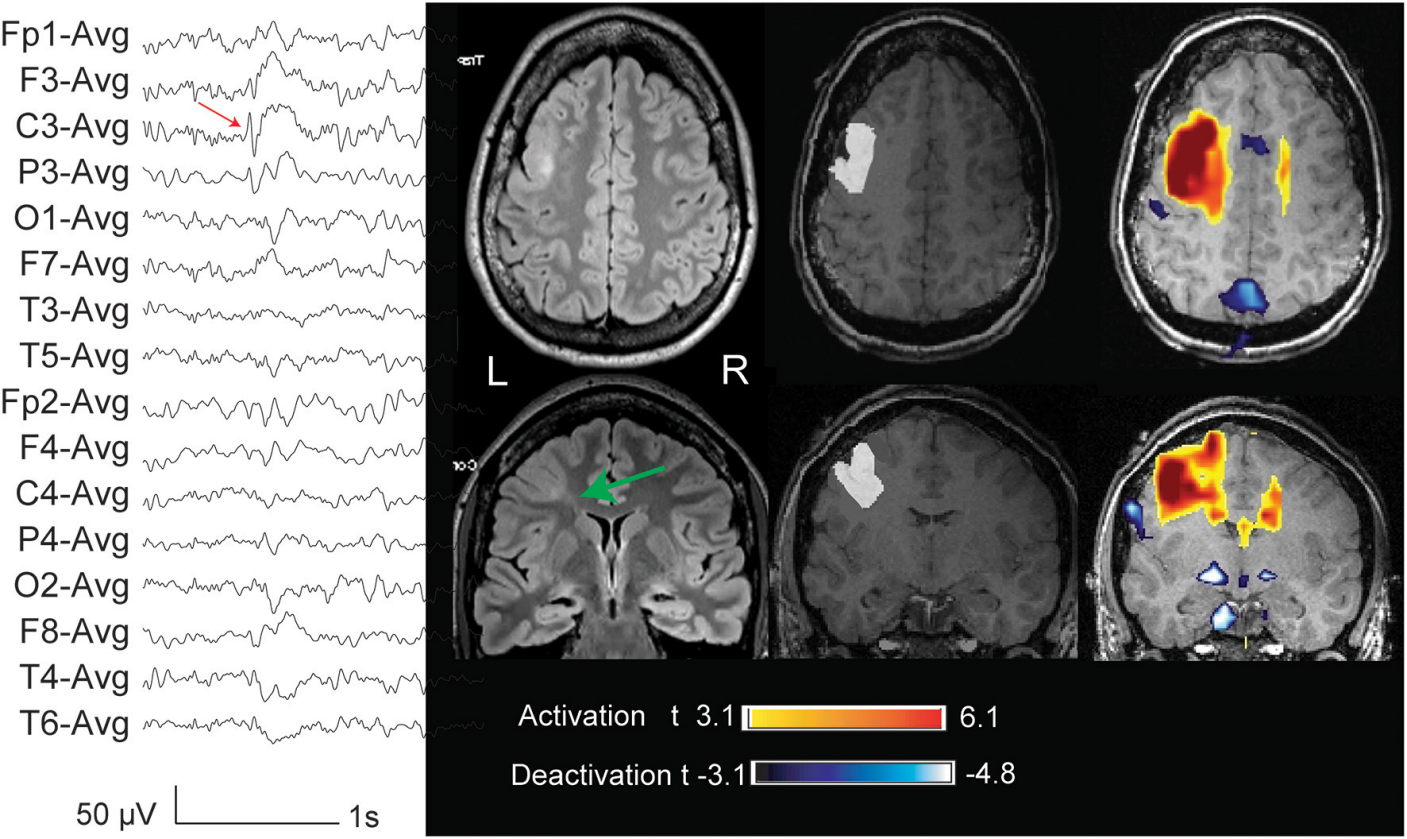
**Thirty two year old patient (case 1) with FCD and macrogyria involving the lips of the middle and inferior left frontal gyri (indicated with a green arrow in the FLAIR sequences acquired outside the EEG-fMRI, and marked in white in the anatomical sequence acquired during EEG-fMRI)**. The marked events were spikes and slow waves maximum at C3 (average reference). The BOLD response showed a focal activation with a maximum *t*-value in the lesion, a cluster in the homologous contralateral region, and deactivation clusters in the precuneus, globi pallidi, and left parahippocampal gyrus.

**Figure 2:**
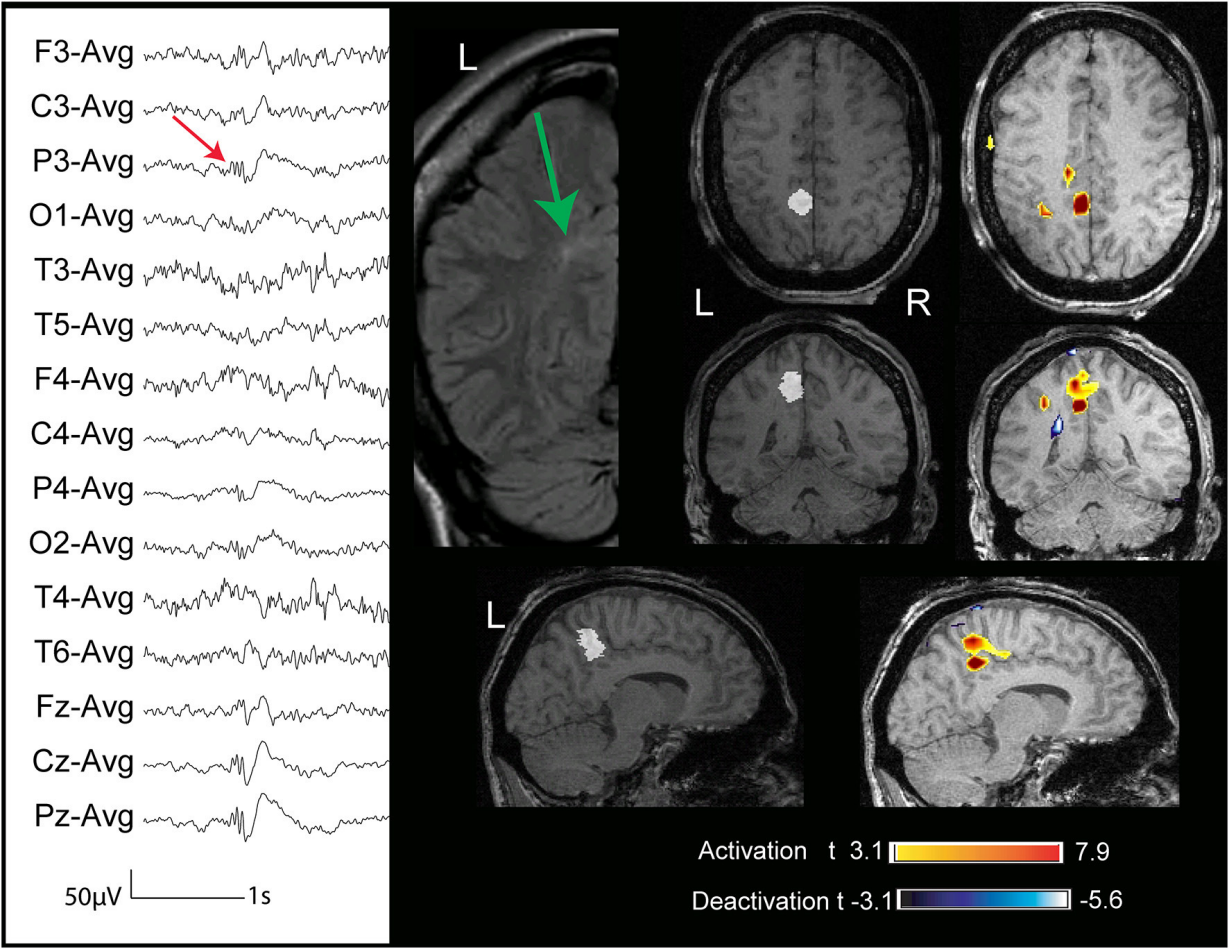
**Thirty four year old patient (case 2) with FCD (marked in white) in the paramedial portion of the left precuneus (the green arrow indicates the lesion in the coronal FLAIR sequence)**. The marked events were polyspikes max at CzPzP3 (average reference). The BOLD response showed a focal activation with a maximum *t*-value in the lesion.

**Figure 3:**
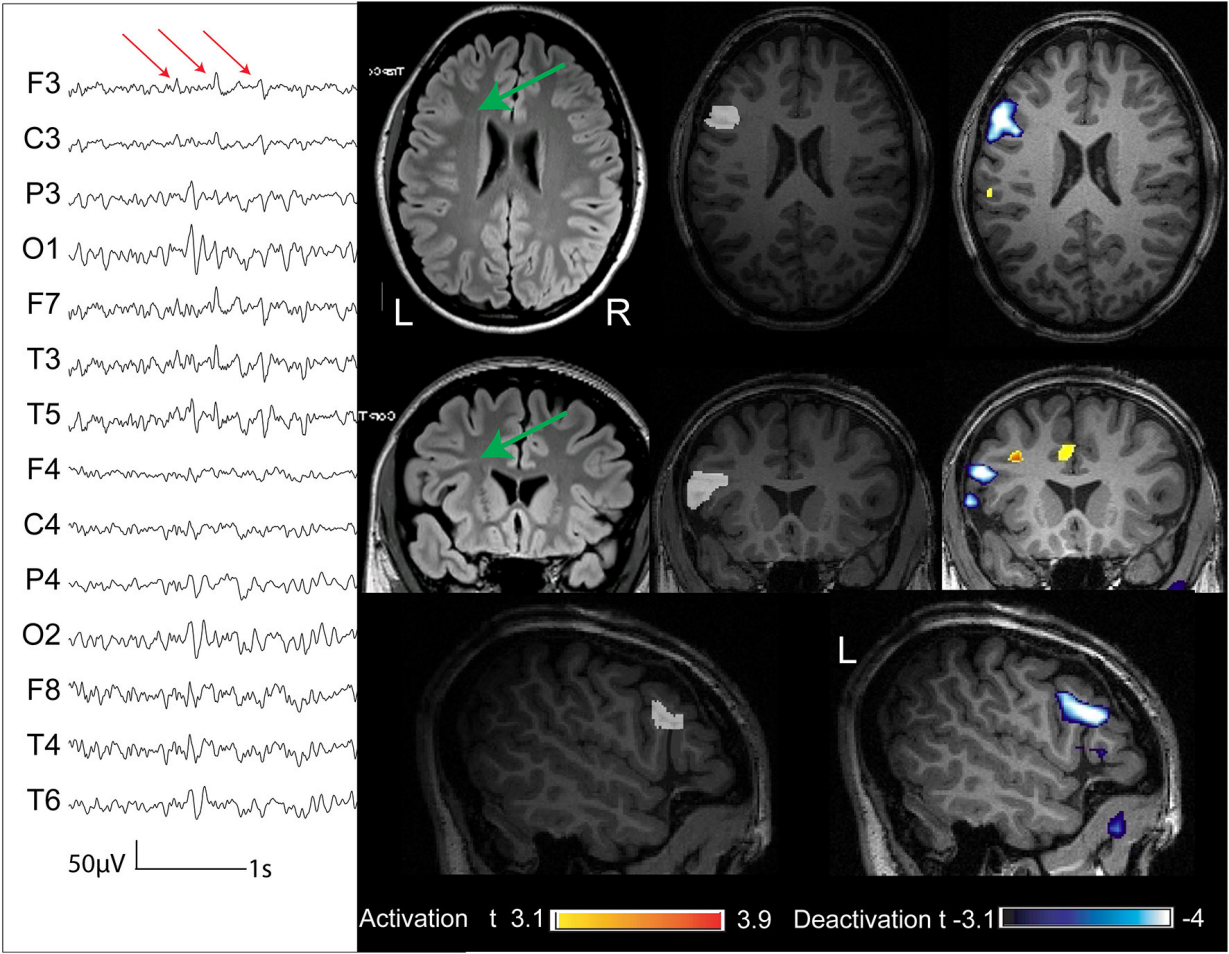
**Twenty six year old patient (case 3) with FCD (marked in white in the anatomical sequence acquired during EEG-fMRI and indicated with a green arrow in the coronal FLAIR sequence) in the L second frontal gyrus**. The marked events were low amplitude spikes max at F3 (referential montage). The BOLD response showed a focal deactivation with a maximum *t*-value in the lesion.

**Figure 4:**
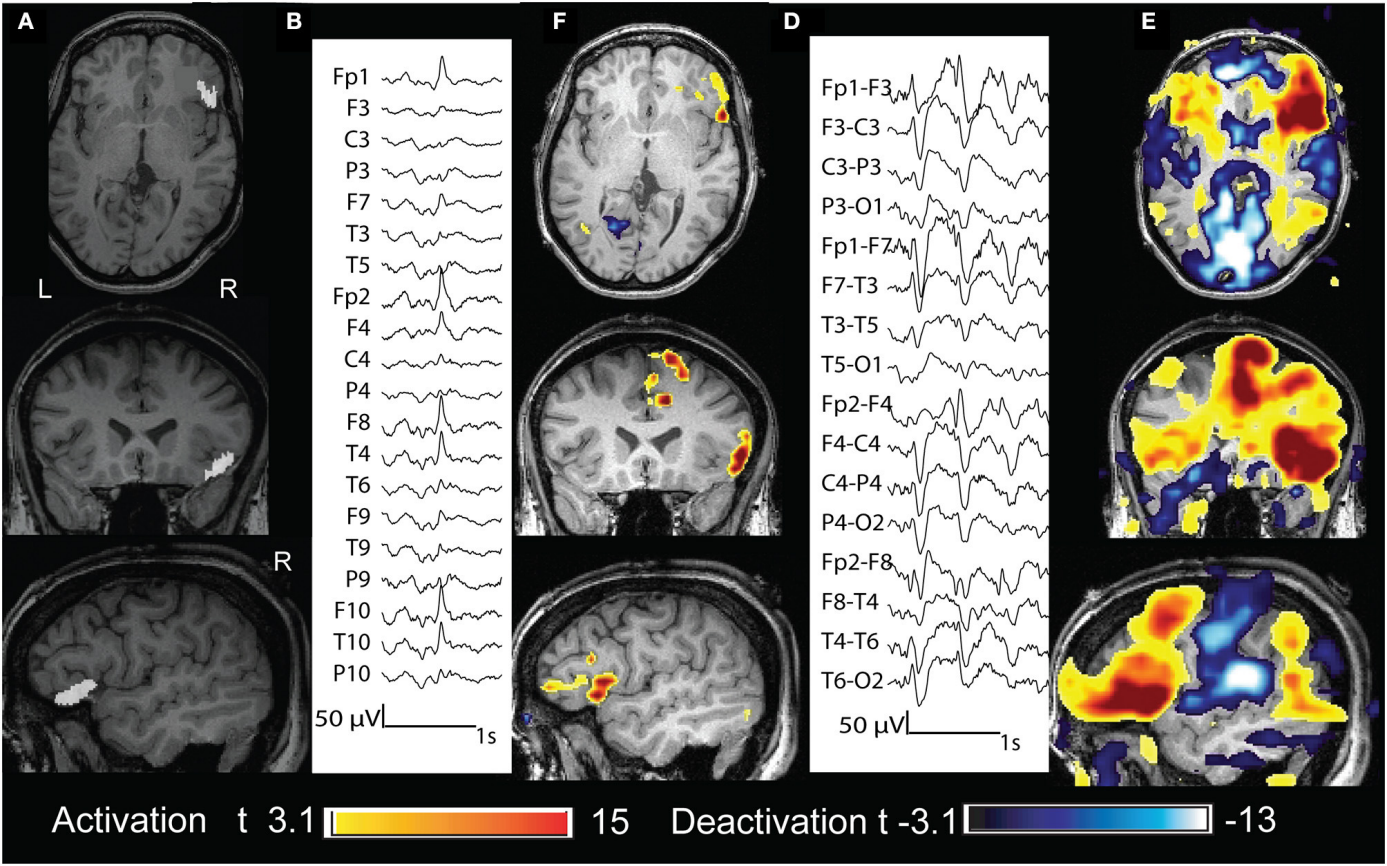
**Twenty three year old patient (case 4). (A)** Structural MRI shows a thickening of the cortical gray matter and blurring of the gray-white matter interface in the right frontal operculum (in white). **(B)** The marked events were spikes with maximum amplitude at Fp2 F8 F10 (reference Fz). **(C)** BOLD response was characterized by different clusters of activation. That with max t value is in the right frontal operculum, corresponding to the lesion. **(D)** The marked events were burst of spike and wave complexes bilateral and diffuse (bipolar montage), but more evident on the right fronto temporal region. **(E)** BOLD response was characterized by different clusters of activation: those with the highest *t*-values were in the right frontal operculum, in the right mid cingulate cortex and cerebellum; clusters of deactivation were present in the areas of the DMN.

**Figure 5:**
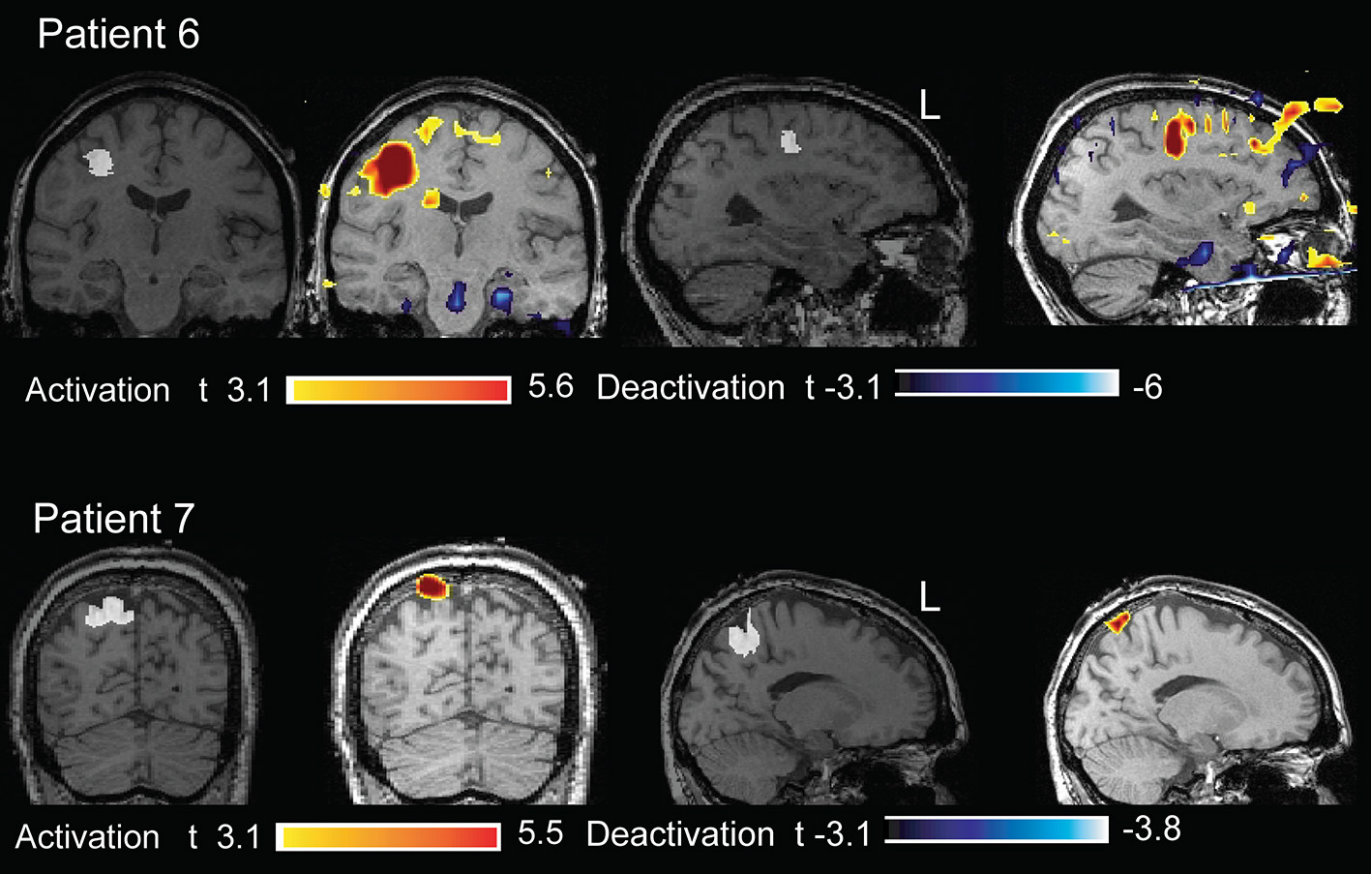
**Two patients (cases 6, 7) whose structural MRI showed a FCD lesion (marked in white on the anatomical acquired during EEG-fMRI after comparison with the FLAIR sequences)**. The BOLD response showed an activation with max *t*-value concordant with the marked lesion.

**Figure 6:**
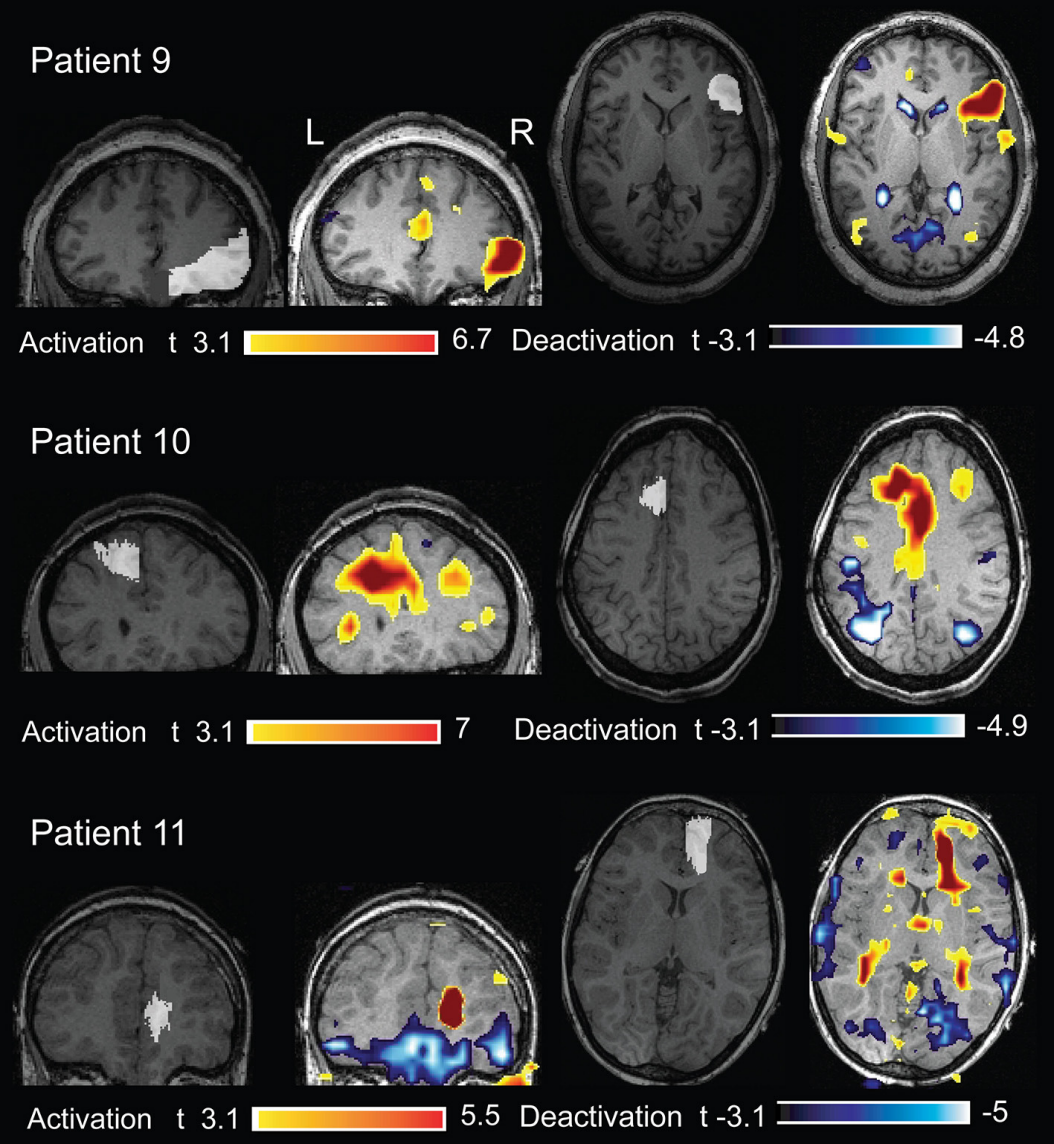
**Three patients (cases 9, 10, and 11) whose structural MRI was negative**. The removed cortex (marked in white on the anatomical acquired during EEG-fMRI after comparison with the post-surgical MRI) showed the presence of FCD. The BOLD response showed an activation with a max *t*-value concordant with the removed cortex.

A BOLD response was observed for each analysis, and it was characterized by both activation and deactivation. In all 13 patients at least one study was concordant with the spike field (Table 2). In 9/13 (69%) patients at least one BOLD response was concordant with the lesion: in 6/7 (86%) MRI+ patients (Figures 1–5) and in 3/6 (50%) MRI− patients (Figure 6). In eight patients the response concordant with the lesion was an activation (Figures 1, 2, 4, 5) and in one a deactivation (Figure 3, Table 2).

**Table 2:** **EEG-fMRI results in the 13 patients showing IEDs during EEG-fMRI; concordance between BOLD, spike field and lesion**.

**Patients**	**Lesion (Volume in mm^3^)**	**IEDs types (number)**	**fMRI max activations (*t*-value) [Volume in mm^3^ of the cluster containing the lesion]**	**fMRI deactivations in the default mode area**	**fMRI max deactivations (*t*-value) [Volume in mm^3^ of the cluster containing the lesion]**	**Concordance between max *t*-value BOLD and spike field**	**Concordance between max *t*-value BOLD and lesion**	**Follow-up Outcome**
**MRI POSITIVE**								
1	L middle and inf frontal gyri (14.151)	C3 spikes (147)	L middle and inf frontal gyri (+8.2) [102.500], lateral ventricles (+6.5), cereb (+6.5), R middle frontal gyrus (+3.5)	Precuneus (−6),L (−8.3) > R angular gyrus	R paracentral region (−6), L (−6.8) >R (−5.8) temporo-parietal junction, bil mesial occipital (−5.3), L (−7) >R (−5.2) globus pallidus, L parahippocampal gyrus (−5.1)	Yes	Yes	N.A.
2	L precuneus (2.419)	Cz-Pz and C3-P3 bursts of polyspikes (29)	L ant precuneus (+6.7) [6.500]	No	White matter above the L occipital horn (−4.5)	Yes	Yes	N.A.
3	L second frontal gyrus (2.382)	F3-F7 low amplitude spikes (459)	L second frontal gyrus (+4), mid L cingulate gyrus(+4.1), L (+4.6)> R (+4.2) posterior cingulate sulcus, L postcentral gyrus (+4.2)	No	L second frontal gyrus (−4.7) [4.364], L (−4.0) > R (−3.8) paramedian first frontal gyrus	Yes	Yes	12 month Engel 1
4	R orbito frontal region (5.190)	Diffuse, but max at F8-T4, spike and slow wave complexes (214)	R frontal operculum (+26) [516.773], L cereb (+28), R mid cingulate gyrus (+26)	Bil precuneus (−16)	Bilateral paracentral regions (−16), mesial occipital (−15), R (−8.8) < L (−11.4) first temporal gyri, R (−5.2) < L (−5.6) hippocampi, L orbitofrontal (−7.5)	Yes	Yes	N.A.
		F8-T4 and F10-T10 spikes (46)	R frontal operculum (+5.4) [5.835], L TO junction (+4.7), R mid cingulate (+4.3), L cereb (+4.2)	No	L cuneus (−4.4), third ventricle (−4.9)	Yes	Yes	N.A.
5	R fronto-polar region (2.359)	Fp2-Fp1-F4-F8 sharp waves (20)	Pons (+5.14)	Bil precuneus (−4.3)	R caudate (−3.8) and R cuneus (−3.6)	No	No	12 month Engel 3
		R > L fronto-temporal regions bursts of rhythmic delta activity (11)	L (+6.3) > R gyrus rectus, R frontal pole (+4.6) [1.928], R (+5.9) > L (+5.5) postcentral gyrus	L precuneus (−4.1)	L sup frontal gyrus (−4.5), L pre-central (−5.0), L post-central (−5.6), L parieto-occipital (−5.7)	Yes	No	N.A.
6	L precentral gyrus (760)	C3 spikes (29)	L precentral gyrus (+7.8) [53.375], L first frontal gyrus (+6.4), L gyrus rectus (+6.1), R cereb (+5)	No	R hippocampus (-5.3), R temporo-occipital region (−6.9), mesencephalon (−5.5)	Yes	Yes	N.A.
7	L parietal lobule (5.177)	C3-P3-O1-Pz spikes (100)	L parietal lobule (+6.2) [3.125]	No	R (−4.2) > L pre-central gyri	Yes	Yes	N.A.
**MRI NEGATIVE**								
8	L sup pre-central gyrus	C3-P3-Cz spikes (34)	L mid postcentral gyrus (+5), L tempor-parieto-occipital (+4.2) R cereb (+3.5), L precuneus (+4.2)	No	R pars triangularis inf frontal gyrus (−4.1)	Yes	No	12 month Engel 4
9	R lateral orbito frontal	F8 spikes (192)	R lateral orbito frontal (+14.8), bil cingulate cortices (+7.2)	No	L (-5.6) > R head caudate, L frontal (−4.4), L (−4.8)>R (−4) cuneus	Yes	Yes	30 month Engel 1
10	L second frontal gyrus	F3-F7 spikes (31)	L second frontal gyrus (+9.9), L (+9.8)> R (+4.8) ant and mid cingulate gyrus, L (+7.8) > R (+5.8) insulae, L thalamus (+5)	R and L angular gyrus (−4.6)	L post-central gyrus (−5.5), L (−6.5) > R (−5.5) temporo-parieto-occipital	Yes	Yes	30 month Engel 4
11	R mesial second frontal gyrus	F4 spikes (297)	R mesial second frontal gyrus (+14). hypothalamus (+8.4), R cingulate (+8.1), L cereb (+6.1) and widespread activation around the lateral ventricles	No	R (−9.8) > L (−7.1) antero mesial orbito frontal region, nuclei accumbens (−8.4), R posterior cingulate sulcus (−7.6), L cuneus (−6.7), R temporal pole (−7.2), R fusiform gyrus (−7.4)	Yes	Yes	N.A.
12	R SMA and mid cingulate cortex	T4 sharp waves (12)	R sup frontal gyrus (+4.9), R caudate (+3.8), R putamen (+3.9), R temporo-parieto-occipital (+4.0)	No	R lingual gyrus (−4.9) and cuneus	No	No	N.A.
		O2-P10 spikes (8)	R fusiform gyrus (+5.5), R mesial frontal gyrus (+3.8), L fusiform gyrus (+4.7)	No	L post-central gyrus (−4.7)	Yes	No	25 month Engel 4
13	L pars opercularis inf frontal gyrus	T3 spikes polyspikes (13)	White matter behind the left occipital horn (+5.2). Surgical bed of the 2 previous resections (+3.8, +4.1)	Precuneus, L and R angular gyrus (−5.6)	L pars orbitalis and triangularis of the inf frontal gyrus (−6.5), L insula (−5.0), L anteromesial margin of surgical bed (−4.8), R frontal operculum (−5.5), R fusiform gyrus	Yes	No	12 month Engel 4

*Ant: anterior. Bil: bilateral. Cereb: cerebellum. IED, interictal epileptic discharge. Inf, inferior. L, left. Post, posterior. R, right. SMA, supplementary motor area. Sup, superior. N.A., not available*.

**Figure 7:**
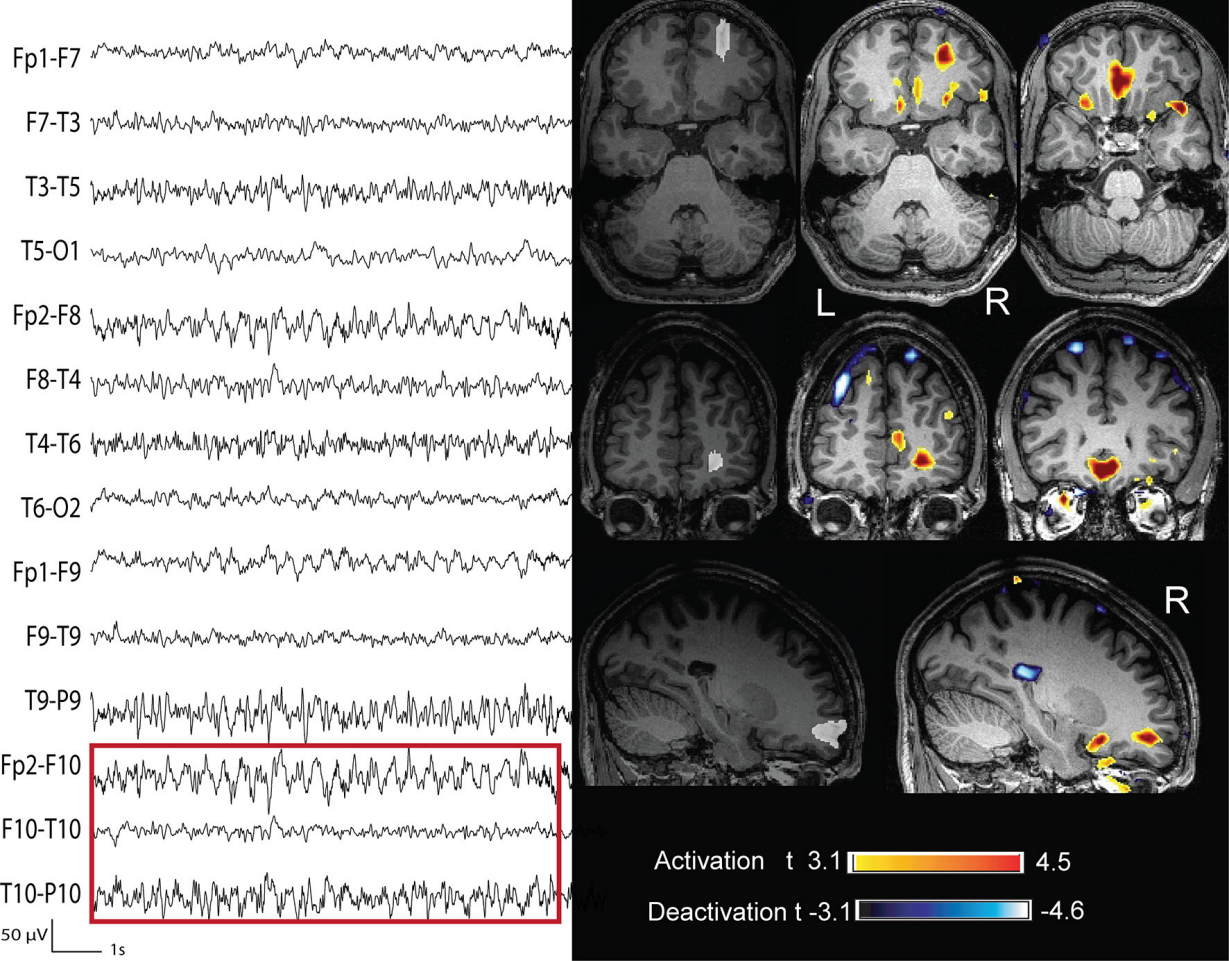
**Thirty year old patient (case 5) with right fronto polar FCD (marked in white)**. The marked event was a prolonged burst of rhythmic delta wave with equipotential at F10-T10 (bipolar montage). The BOLD response was characterized by a cluster with max *t*-value on both giry recti and an activation with a lower *t*-value in the area subsequently removed. We considered this response as not concordant.

**Figure 8:**
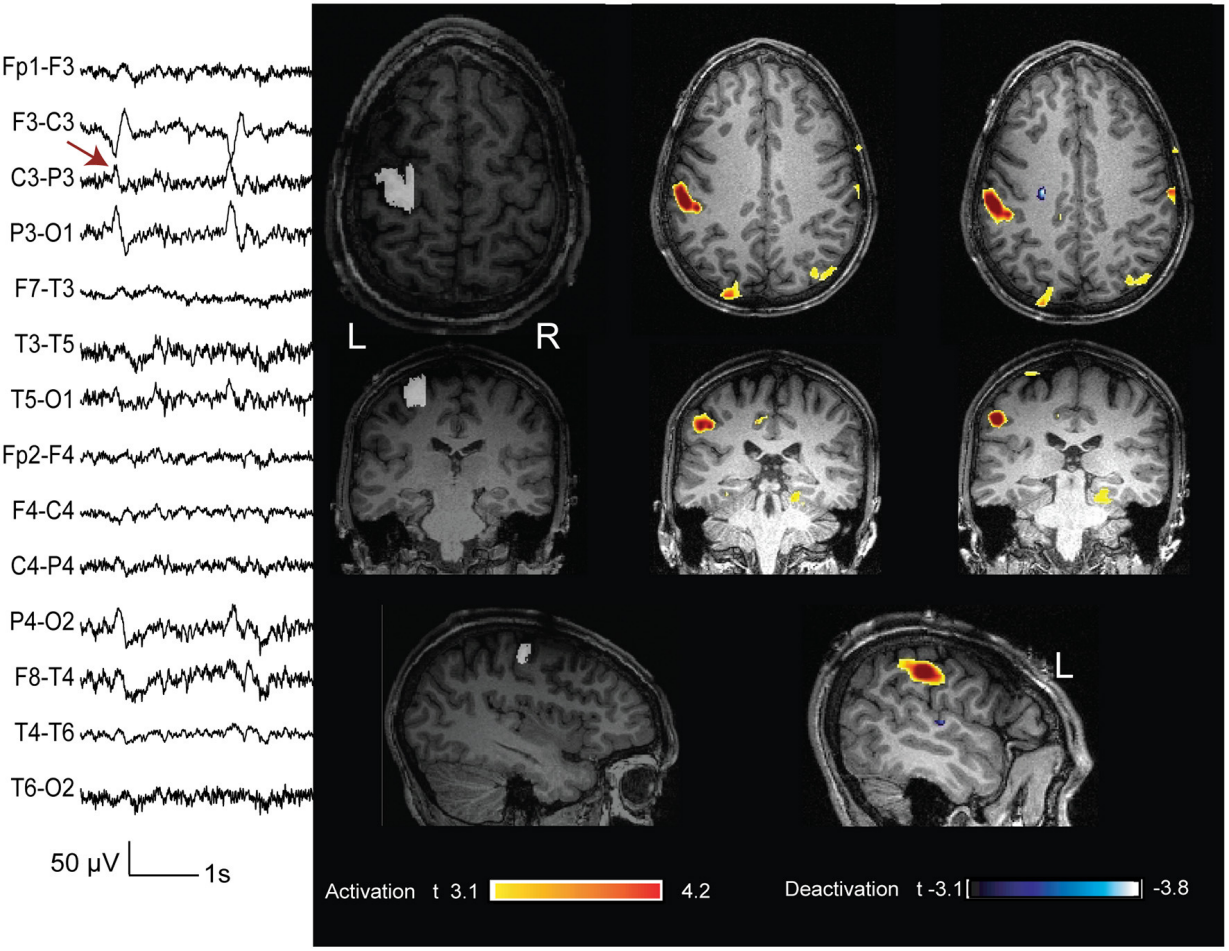
**Twenty year old patient (case 8) with seizures characterized by a tingling sensation starting in the right hand digits and then propagating to the forearm, followed by right arm stiffness and jerks and sometimes secondarily generalization**. The structural MRI did not show any lesion. The marked events were spikes with phase reversal at C3 (bipolar montage). The BOLD showed different clusters of activation: that with the highest *t*-value is in the postrolandic mid-sensory cortex. The intracerebral chronic electrocorticogram confirmed this finding. During surgery the pre-central gyrus was removed (white marking) and the histopatology revealed the presence of dysplastic cells. This case was considered as not concordant.

**Figure 9:**
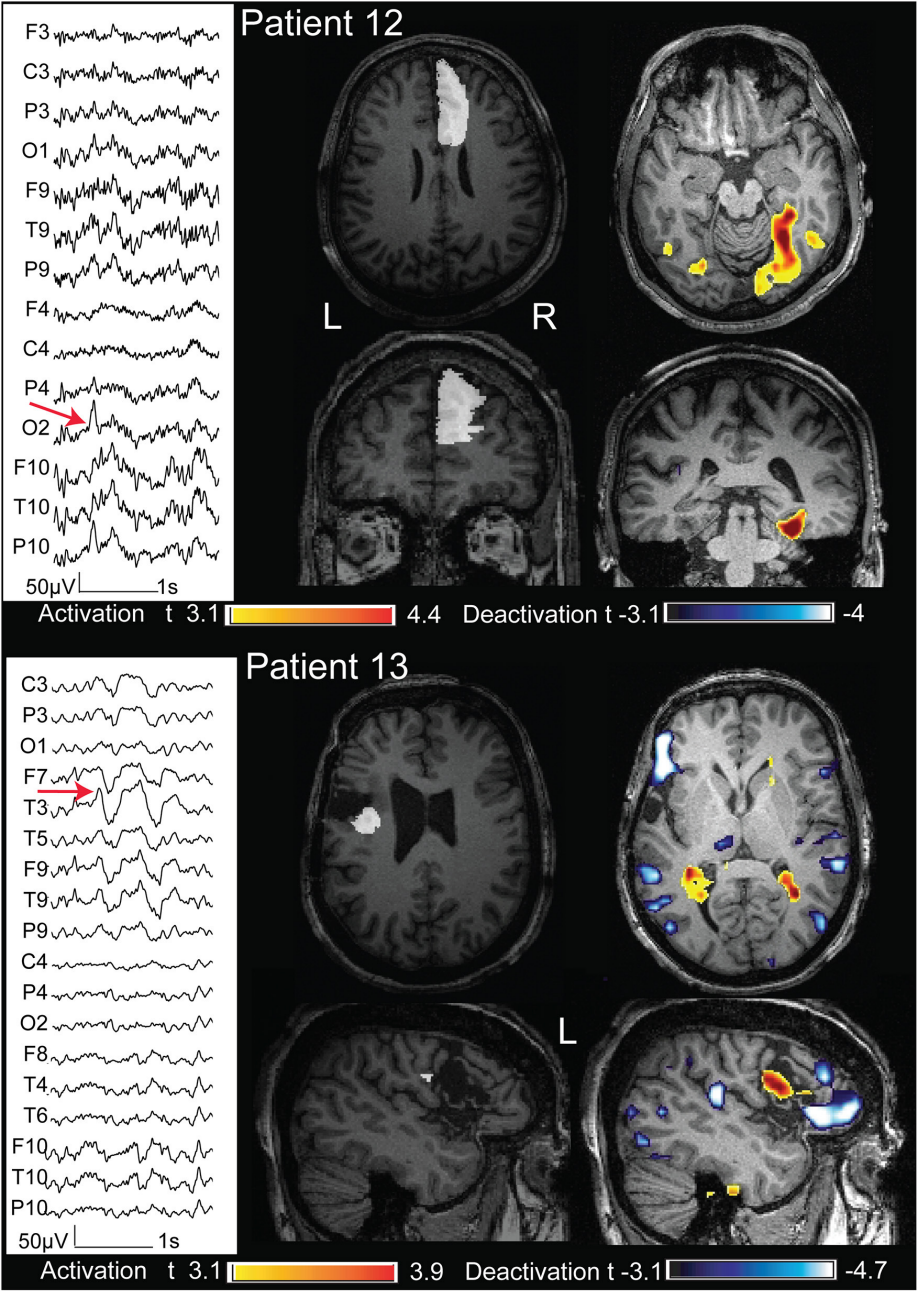
**Above: case 12**. The marked events were spikes max at P10 O2 (the electrode T6 had an artifact during the acquisition). The BOLD response showed an activation with max t value in the right fusiform gyrus. The removed cortex cointaining the FCD was the right supplementary motor area (marked in white). Below: case 13. The marked events were T3 spikes. This patient had three operations and the surgical bed is evident on the structural MRI. The cortex removed during the last operation (marked in white) showed the presence of a FCD. The BOLD response showed a max deactivation more anterior than the removed cortex. These 2 cases are not concordant.

In 10/16 (62%) fMRI studies, concordance was found between highest BOLD response and lesion (in 9 the highest *t*-value was inside the lesion or removed cortex and in 1 it was within 1 cm from the border of the resected cortex). In six studies the BOLD response with max *t*-value was not concordant with the lesion/resection: patient 5 (MRI+, 2 studies) with the MRI lesion located in the right fronto-polar region and the two analyses showing the highest activation in the pons and gyrus rectus bilaterally (Figure 7); patient 8 (MRI−, one study) had the BOLD max *t*-value in the left post-central cortex, but the resection was just anterior to the motor cortex (Figure 8); patient 12 (MRI−, 2 studies) had one study with the max *t*-value localized in the right fusiform gyrus (Figure 9) and the other with the highest *t*-value in the right superior frontal gyrus, and in this patient the right supplementary motor area was resected; and, finally, in patient 13 (MRI−, one study), the max *t*-value was in the left anterior frontal lobe but more anterior than the area resected (Figure 9).

In five of the seven MRI+ patients, the volume of the BOLD cluster concordant with the lesion was larger than the lesion itself (Table 2). In six of seven patients this cluster had the highest *t*-value of the analysis (six concordant MRI+ patients).

In 15 studies, concomitant cortical activation clusters with a lower *t*-value were seen remotely from the lesion: in two the activation was in the contralateral homologous area, and in the others they were either in the same or in different lobes. Nine studies had activation clusters in subcortical structures: five in the cerebellum, one in the pons, one in thalami, one in putamen, and both caudate nuclei, one in hypothalamus, and cerebellum.

Deactivation cortical clusters were also observed in all 16 studies, remotely from the lesion: in six cases deactivation responses were present in brain regions belonging to the default mode network, in the others they were in different lobes. Six studies had deactivation clusters in subcortical structures: two in caudate nucleus, one in mesencephalon, and hippocampus, one in hippocampus, one in globus pallidus, and one in nucleus accumbens.

## Discussion

This study describes the EEG-fMRI findings of patients with focal epilepsy and FCD. The first finding is that all patients (13/13) showing an active EEG during the acquisition had a BOLD response (activation and deactivation). This high ratio is concordant with a previous 3T study (Thornton et al., 2011) where 92% of the patients (11/12) also showed significant spike-related hemodynamic changes. Although we cannot exclude that these results are explained by a higher sensitivity of 3T EEG-fMRI compared to 1.5T, as expected from higher magnet strength (Kruger et al., 2001; Gholipour et al., 2011; Grouiller et al., 2016), this high rate of responses could be due to the high degree of epileptogenicity of FCD (Palmini et al., 1991; Gambardella et al., 1996). This intrinsic high epileptogenicity has been related to the malfunction of GABA-mediated inhibition in the dysplastic neocortex (Spreafico et al., 1998; Avoli et al., 1999; Sakakibara et al., 2012; Medici et al., 2016) and in peri-lesional tissue (Hodozuka et al., 2006), as demonstrated by electrophysiological and histochemical studies. MR spectroscopy (MRS)/BOLD studies have also shown an inverted relationship between the resting state GABA concentration, and amplitude of BOLD responses (Muthukumaraswamy et al., 2012). Hence, the high number of BOLD responses found in patients with FCD may be related to the decreases or malfunction of GABA-mediated inhibition described in the brain of the patients with this type of lesion.

This study showed overall a good concordance between localization of the highest BOLD response and localization of the lesion: this was found in 69% of the patients that had an active EEG during acquisition. In patients with a lesion detectable by structural MRI, concordance was excellent and found in 86%, but EEG-fMRI was also able to localize the lesion in 50% of patients with negative structural MRI. In such MRI− cases, the location of BOLD responses could be used to help the detection of occult lesions by targeted revision of MRI studies or to guide intracranial electrode implantation in patients with focal epilepsy likely explained by an invisible FCD, after integration with information from the clinical context and other diagnostic techniques (Guerrini et al., 2015).

In our series, just 8 of 13 included patients underwent surgical treatment; indeed often the epileptogenic zone was located in eloquent areas. Although with very small numbers, we can observe that the operated cases (one MRI+ and one MRI−) who were seizure free at >12 months of follow up, had a BOLD response concordant with the lesion. Concerning patients who kept having seizures (one MRI+ and 4 MRI−), the BOLD was not concordant to the resected area in 4/5 cases, although it was concordant with the spike field in all but one. This finding, although on small number, strengthens the concept of negative predictive value, as expressed by An et al. (2013) and Coan et al. (2016). The design of our study allowed us indeed to detect the presence of FCD in the areas that were surgically removed, but does not allow us to assess if the areas where we observed a BOLD responses contain FCD. This can be assumed at least in some cases, particularly because all the BOLD responses were concordant with the spike field. For example in patient 8, seizure semiology and EEG data obtained from a cortical grid were highly suggestive of a post-central focus, and the EEG-fMRI study from spikes with phase reversal at C3-P3 showed the highest BOLD response in that region. However, during surgery the pre-central gyrus appeared macroscopically abnormal and was removed, and the histopathology revealed FCD type IIb. The patient was not seizure-free after the resection, suggesting a more widespread lesion and a postcentral gyrus possibly also containing dysplastic tissue. Another example is patient 12, in whom intracerebral EEG revealed multifocal interictal epileptic activity arising from the right supplementary motor area (SMA) and right posterior hippocampus. The semiology and ictal EEG activity supported an early SMA involvement, and this region was resected showing a type Ia FCD. The BOLD response with the highest *t*-value was however obtained from the posterior quadrant spikes, with activation in the right fusiform gyrus. This patient is not seizure-free, and the EEG-fMRI findings may have again suggested a diffuse or multifocal cortical dysplasia. Previous studies showed that FCD may be associated with multiple areas of epileptogenicity (Chassoux et al., 2000; Fauser et al., 2009), some appearing structurally normal. An EEG-fMRI study correlating spike-related hemodynamic changes, intracranial EEG findings and surgical outcome (Thornton et al., 2011) supports this evidence.

Our results also suggest that the BOLD response, and hence the metabolic demand, is not dependent on the histological FCD subtype. Eight patients were operated and type I was found in one (MRI−), type IIa in two (also MRI−) and type IIb in five (2 MRI+ and 3 MRI−).

The BOLD responses with the highest t value were activations in 14 analyses, and deactivations in two. In one patient (patient 3), the negative BOLD response was concordant with the spike field and with the lesion, and in another (patient 13), although the BOLD was concordant with the spike field, it was not concordant with the lesion. This is in agreement with previous EEG-fMRI studies in FCD patients that found deactivations in the epileptic focus in only a small proportion of cases (Federico et al., 2005; Thornton et al., 2011). The localizing value of the negative BOLD is still debated and its significance is not completely clarified (Kobayashi et al., 2006a). Indeed localizing deactivation has been associated in a few cases with an earlier activation (Rathakrishnan et al., 2010); in other cases, it can occur in the region of IED generation, mostly if such IEDs are accompanied by high amplitude slow waves (Pittau et al., 2013). In our study, we distinguished the deactivations belonging to the DMN from those with a possible localizing value in order to divide responses coming from a network inhibition from those useful to the localization of the focus. A deactivation in the areas of DMN was present in 38% of the analyses; as demonstrated by other studies, this proportion is less consistent than the DMN deactivation present in other type of common pathology, as mesial temporal lobe epilepsy (Laufs and Duncan, 2007; Coan et al., 2016), most probably because HRF peak amplitude in deactivation clusters is larger in the mesial temporal lobe epilepsy group than in the FCD, when the deactivation occurred in DMN regions (Watanabe et al., 2014).

In this study, we found that BOLD responses are generally much larger than the visible lesion. We observed that in only one study (patient 2) the hemodynamic response was very focal and coinciding with the FCD; even in this case the volume of the BOLD cluster concordant with the lesion was bigger than the visible anatomical anomaly. This discrepancy between the volumes of the anatomical lesion and the BOLD cluster containing the lesion is present in almost all cases, and it could depend on the rate of IEDs or on the statistical models used for the analysis. However, the same phenomenon is common in another technique used to localize interictal focus, i.e., Positron Emission Tomography (PET). The focal interictal hypometabolism of radio-labeled fluoro-deoxy-glucose-PET is usually larger than the epileptogenic cortex, reflecting probably the altered function not only of the interictal focus, but also of the areas involved by the first ictal spread (Nelissen et al., 2006; Pittau et al., 2014a). In all the other studies, remote cortical or subcortical structures were activated suggesting that focal IEDs recorded from scalp EEG may represent only a fraction of broader metabolic events that involve widespread epileptic network (for review: Pittau et al., 2014b). These findings are in agreement with previous studies on focal epilepsy from malformations of cortical development (Kobayashi et al., 2006b) or from heterogeneous etiology (Fahoum et al., 2012). These findings are strengthened by other localizing technology, as stereo-EEG (SEEG): Varotto et al. (2012) in a series of patients with type II FCD studied with SEEG demonstrated a specific connectivity pattern (mainly in the gamma band) between the epileptogenic zone and other distant cortical regions during the ictal, inter- and pre-ictal periods. They suggested that the lesional nodes act as the center of the epileptic network where seizures originate and are sustained, and that the cortical regions beyond the dysplasia, also involved in the ictal activity, essentially act as “secondary” generators of synchronous activity.

A limitation of our study is that concordance between EEG and BOLD has been established by visual analysis. Electric source analysis (ESI) is an alternative and precise method to localize interictal spikes, when obtained from an electrode setup of at least 64 electrodes (Lantz et al., 2003). Indeed it has been shown (Brodbeck et al., 2011) that ESI has a high sensitivity (84.1%) and specificity (87.5%) on localization precision when obtained with high-resolution electric source imaging (>128-electrodes) and individual MRI as the head model. However, in the same study, sensitivity and specificity decreased to 66 and 54%, respectively when EEG was recorded with a low number of electrodes (19–29 channels). Studies combining high density EEG and fMRI will solve this limitation (Vulliemoz et al., 2009).

It is known that electrodes placed inside a FCD usually show almost continuous spiking activity (Palmini et al., 1995), but on scalp EEG much less frequent spikes are detected. How is it possible that these scalp spikes also demonstrate a source that appears to be in the FCD? One can speculate that occasionally the dysplastic focus generates a widespread discharge that reaches the scalp, and that at the time of this discharge the metabolic activity is even more intense in the dysplasia than during “background” more localized spiking, thus generating a maximal BOLD response in the dysplasia.

To conclude, our study shows a high level of concordance between FCD and BOLD response. In some MRI-negative cases the BOLD response did not correspond to the resection, but we provided evidence that the region of BOLD response could nevertheless correspond to dysplastic and epileptogenic tissue. This could be useful especially for MRI negative patients, where the location of BOLD responses can be used to detect occult lesions by targeted revision of MRI studies, or for guiding intracranial electrode implantation. However, in almost all FCD patients, a metabolic involvement of remote cortical or subcortical structures was also found, corroborating the idea that focal IEDs recorded from scalp EEG may represent only a fraction of broad metabolic events that involve a widespread epileptic network in spite of their focal appearance.

## Ethics statement

This study was carried out in accordance with the recommendations of Neurosciences Panel of the MUHC Research Ethics Board with written informed consent from all subjects. All subjects gave written informed consent in accordance with the Declaration of Helsinki. The protocol was approved by the Neurosciences Panel of the MUHC Research Ethics Board.

## Author contributions

FP: Substantial contributions to the conception or design of the work. Recording and analysis EEG-fMRI data. Writing the manuscript. Revision and interpretation data. LF: Substantial contributions to the conception or design of the work. Analysis EEG-fMRI and making figure. FF: Recording and analysis EEG-fMRI data. Revising it critically for important intellectual content; FD: recruiting patient. Revision and interpretation data. Revising it critically for important intellectual content; JG: idea of the project. Revision and interpretation data. All authors: Agreement to be accountable for all of the work in ensuring that questions related to the accuracy or integrity of any part of the work are appropriately investigated and resolved. Final approval of the version to be published.

## Funding

This work was supported by the Canadian Institutes of Health Research (CIHR) grant MOP-38079. FP was supported by the Savoy Foundation for epilepsy.

### Conflict of interest statement

The authors declare that the research was conducted in the absence of any commercial or financial relationships that could be construed as a potential conflict of interest.
